# Performance factors that negatively influence shooting efficiency in women’s basketball

**DOI:** 10.3389/fphys.2022.1042718

**Published:** 2022-11-25

**Authors:** Tomáš Vencúrik, Zoran Milanović, Anja Lazić, Feng Li, Kęstutis Matulaitis, Tomislav Rupčić

**Affiliations:** ^1^ Faculty of Sports Studies, Masaryk University, Brno, Czech Republic; ^2^ Faculty of Sport and Physical Education, University of Niš, Niš, Serbia; ^3^ Science and Research Centre of Koper, Koper, Slovenia; ^4^ China Basketball College, Beijing Sport University, Beijing, China; ^5^ Department of Coaching Science, Lithuanian Sports University, Kaunas, Lithuania; ^6^ Laboratory for Sports Games, Faculty of Kinesiology, University of Zagreb, Zagreb, Croatia

**Keywords:** basketball shooting, team sports, coaching, logistic regression, performance analysis

## Abstract

The purpose of the present study was to examine the influence of selected factors (possession duration, game quarter, defensive pressure, shooting distance from the basket, and heart rate level) on shooting efficiency on Under-19 (U19) and senior level of women’s basketball competition (second division). The analysis procedures included five U19 and six senior-level games, containing 224 and 252 shooting attempts, respectively. Binary logistic regression identified the opponent’s defensive pressure and shooting distance from the basket as significant predictors of shooting efficiency in both categories. When defensive pressure was high, the chance for the missed shot was 3.5 (95% CI; 1.43–8.52) and 3.19 (95% CI; 1.4–7.26) times more likely than it was under the minimum defensive pressure for U19 and senior category, respectively. Shooting efficiency significantly decreased when the horizontal distance from the basket increased. In U19, a chance for a missed shot was 4.63 (95% CI; 2–10.712) and 5.15 (95% CI; 1.91–13.86) times higher for medium and long-distance (respectively), compared to short-range shooting. In the senior category, the chance for the missed shot was 3.9 (95% CI; 1.83–8.31) and 3.27 (95% CI; 1.43–7.52) times higher for medium and long-distance (respectively) when compared to a short distance. The possession duration, game quarter, and heart rate level were identified as insignificant predictors. Therefore, the aforementioned findings suggest that basketball players and coaches may benefit from designing training sessions where the defender puts pressure on the shooting player as in a real game situation and balanced the frequency of shooting from different distances from the basket.

## Introduction

Basketball is an intermittent, and a complex game where shooting is one of the most important technical skills of the game (Zwierko et al., 2018; Li et al., 2021). More precisely, shooting efficiency is considered one of the crucial performance indicators due to the strong positive relationship between field goal shooting and winning the game (Ibáñez et al., 2009; Lorenzo et al., 2010; García et al., 2013; Cabarkapa et al., 2022b). However, shooting performance in a game can be determined by various endogenous and exogenous factors that disrupt its effectiveness. It is well known that basketball players have to perform a shot under various conditions (e.g. internal and external loads, fatigue) (Pojskic et al., 2018). Previous studies (Ardigò et al., 2018; Padulo et al., 2018) have confirmed a decrease in the successfulness of field goal shooting of male players under a high-intensity condition compared to a medium intensity condition and at rest. Additionally, due to high intensity and accumulated fatigue, a change in the biomechanical parameters of the shooting may consequently disrupt the shooting performance (Erčulj and Supej, 2009).

In addition to the previous findings (Llorca-Miralles et al., 2013), when players choose to shoot in a game, they mainly consider four factors: defensive pressure, rebounding issues, defensive balance, and shooting distance. Within this area of investigation, there is a strong evidence that the presence of a defender can affect the shooting performance of male players (Rojas et al., 2000; Csataljay et al., 2013). Specifically, the presence of a defender modifies the speed and the height of ball release during two-point (2 PT) shooting, which may also be related to a decrease in shooting efficiency. Moreover, with an increase in defensive pressure (the distance between the offensive and the defensive player decreased), the probability of successful shooting outcome decreases.

Subsequently, most researchers in the field of shooting performance (Miller and Bartlett, 1996; Okazaki and Rodacki, 2012; Vencúrik et al., 2021a) also drew parallels between shooting efficiency and the distance from the basket in male basketball players. More precisely, with increasing horizontal distance, segmental joint angles, a center of mass displacement, release angle of the ball, entry angle of the ball, and release speed of jump shot changed significantly (Okazaki and Rodacki, 2012; Cabarkapa et al., 2022a; 2022c). The shooting efficiency was also reduced, with players having to reorganize the coordination of body segments to meet the requirements of the new motor task (Okazaki and Rodacki, 2012; Vencúrik et al., 2021a).

Most scholars seem to agree that various factors strongly determine basketball shooting performance. However, to date, scant attention has been paid to the influence of these factors during the official games. Moreover, previous research has largely overlooked the role of disruptive factors in female basketball players. Within the field of investigation, a number of crucial questions remain unanswered. For this reason, this study aims to identify disruptive factors that can predict the shooting performance of female basketball players in real game conditions. We hypothesized that selected factors would affect shooting efficiency (heart rate level, possession duration, game quarter, defensive pressure, and shooting distance from the basket).

## Materials and methods

### Participants

Twenty-six female basketball players participated in the research. Fourteen young elite players were from the first division of the U19 age category in the Czech Republic, and twelve semi-elite players were from the second division of the senior category in the Czech Republic. Because some players did not meet the inclusion criteria (15 min played and start in both halves), their data of shooting efficiency were not processed. In addition, the researchers could not influence the playing time of the players because these were competitive games. Finally, data from eighteen female basketball players were processed. Ten young elite players were from the first division of the U19 age category (age: 17.6 ± 1 year; body height: 179.4 ± 6.2 cm; body mass 62.9 ± 5.3 kg), and eight semi-elite players were from the second division of the senior category (age: 20 ± 2.8 years; body height: 179.8 ± 4.9 cm; body mass 66.8 ± 5.7 kg). During the weekly microcycle, all players had four to five training units (strength and conditioning, technical and tactical). Players of both categories played two games every other weekend.

Players signed informed consent (or their legal representative) to voluntarily participate in the research. The study was conducted in accordance with the Declaration of Helsinki and according to the ethical standards of Masaryk University (ethical statement EKV-LS-2020–003).

### Procedure

Shooting performance was monitored in the U19 category in five competitive games and in the senior category in six competitive games (regular season games). Games were streamed online and were freely available on the internet server. All games were played according to the International Basketball Federation (FIBA) rules and were officiated by two experienced referees. The court had standard dimensions 28 × 15 m, and playing time was divided into four 10-min quarters. The half-time break lasted 15 minutes, breaks between first and second quarters and between third and fourth quarters lasted 2 minutes.

At the beginning of the research (2 weeks before first game), the players completed a beep test, in which their maximum heart rate (HR_max_) was recorded at the end of the test (Ruiz et al., 2009). The players’ heart rate (HR) was monitored using a commercially available Suunto Team device (Suunto Oy, Vantaa, Finland). HR sensors (Suunto Memory Belts) were placed below the chest level, measured HR in 2-s intervals and were synchronized with the playing time. The HR data were evaluated using the software Suunto Training Manager (Suunto Oy, Vantaa, Finland).

Dartfish TeamPro 6.0 software (Dartfish, Friborg, Switzerland) was used for notational analysis. The shooting attempt was considered successful if the player scored or was fouled in the attempt (Gómez M. et al., 2015). An unsuccessful shooting attempt was considered if the player did not score or the defender blocked the shot. In total, 224 shooting attempts in the U19 category and 252 shooting attempts in the senior category were recorded and evaluated. Each shooting attempt (dependent variable) was assigned a disruptive factor (independent variable) value, which could affect its efficiency. Based on the previously published literature (Refoyo et al., 2009; Álvarez et al., 2009; Ben Abdelkrim et al., 2010; Vaquera et al., 2013, 2016; Gómez M.-Á. et al., 2015; Gómez et al., 2015 M.; Courel-Ibáñez et al., 2016; Vencúrik et al., 2021b) we distinguished five disruptive factors: defensive pressure, ball possession duration, game quarter, shooting distance, heart rate level (Table 1). The defensive pressure on the shooting player was classified as: 1) low or no physical pressure on the shooting player, 2) moderate physical pressure on the shooting player, 3) high physical pressure on the shooting player (Refoyo et al., 2009; Álvarez et al., 2009; Gómez M. et al., 2015). Due to the intermittent nature of the activities, there is a lag response in HR; and it possesses some inaccuracies (Berkelmans et al., 2018). The HR exponential decrease starts about 10 s later than the end of the sprint (Storniolo et al., 2020). Therefore, each shooting attempt was assigned an average HR value ranging from -5 s to +10 s from the moment the ball was released. The raw data can be found in the Supplementary Material.

**TABLE 1:** Performance and game indicators observed in relation to shooting efficiency (i.e., made, missed).

Variable		Description
Performance and game indicators	Heart rate level	<85% HR_max_
		85–95% HR_max_
		>95% HR_max_
	Possession duration	0–8 s
		9–16 s
		17–24 s
	Game quarter	First
		Second
		Third
		Fourth
	Defensive pressure	Low
		Moderate
		High
	Shooting distance	Short (<2.5 m)
		Medium (2.5–6.75 m)
		Long (>6.75 m)

Because the notational analysis method was used, it was necessary to ensure the objectivity (inter-rater agreement) and reliability (intra-rater agreement) of the evaluation for the independent variables of the defensive pressure and shooting distance (O’Donoghue, 2015). Three independent expert observers ensured objectivity by evaluating a total of 48 randomly selected defensive pressure situations and 48 randomly selected shooting distance situations (10% from all shooting situations). Observers had to meet the following criteria: 1) at least 10 years of coaching experience, 2) at least 10 years of experience as a university researcher or lecturer, 3) the highest national coaching license, 4) the international FIBA Coaching License. Observers were trained and instructed on the evaluation procedure.

The reliability (intra-rater agreement) was ensured by repeated evaluation of the same 48 randomly selected defensive pressure situations and 48 randomly selected shooting distance situations. The situations were assessed at two different time points, 12 weeks from each other. This time interval was chosen, so the observer did not remember the individual game situations.

### Statistical analysis

The independent variables of defensive pressure and shooting distance were of the ordinal type; therefore, objectivity (inter-rater agreement) and reliability (intra-rater agreement) of their evaluation were determined by Krippendorff’s alpha (KALPHA) and its 95% confidence intervals (CI). The KALPHA can take values from 0 to 1, where: 1 = perfect agreement; 0.99–0.8 = good agreement; 0.79–0.67 = acceptable agreement; bellow 0.67 = poor agreement (Hayes and Krippendorff, 2007).

The relationship between the dependent variable and the independent variables was expressed by Pearson’s chi-square test. The effect size was calculated by the Creamer contingency coefficient (V) and interpreted as: 0.1 = small effect; 0.3 = medium effect; 0.5 = large effect (Volker, 2006).

The binary logistic regression method was used to predict the shooting efficiency based on independent variables (Malek et al., 2018). This means that the execution of the shooting could acquire only two values, i.e., 0–ineffective shooting or 1–effective shooting. The maximum likelihood method was used to estimate the regression coefficients. The independent variables were specified as categorical and were replaced by dummy variables. The reference category was set for each independent variable. For heart rate level, it was category <85% HR_max_; for possession duration, it was category 0–8 s; for the game quarter, it was the category first quarter; for defensive pressure, it was the category low pressure; and for shooting distance, it was the category of short distance (up to 2.5 m). The likelihood ratio was then expressed with relation to the reference category (Malek et al., 2018). A logistic regression model that best describes the data was sought using backward stepwise selection. Wald’s test verified the statistical significance of the regression coefficients, and 95% confidence intervals were constructed for the likelihood ratio. The Hosmer-Lemeshow goodness of fit test determined the differences between the observed and expected frequencies. A statistical significance was set for *α* = 0.05. Statistical data processing was performed in IBM SPSS Statistics 25 statistical software (IBM Corp., Armonk, NY, United States).

## Results

### Objectivity and reliability

Table 2 shows the inter-rater agreement and intra-rater agreement in evaluating the independent variables of defensive pressure and shooting distance. All calculated KALPHA values indicate a good agreement.

**TABLE 2:** Inter-rater and intra-rater agreement in defensive pressure and shooting distance.

Agreement	Defensive pressure		Shooting distance	
	KALPHA	95% CI	KALPHA	95% CI
Inter-rater agreement	0.87	0.79–0.94	0.97	0.92–1
Intra-rater agreement	0.88	0.74–0.99	0.97	0.92–1

Note: KALPHA, Krippendorff’s alpha; CI, confidence interval.

### Relation between variables

**FIGURE 1:**
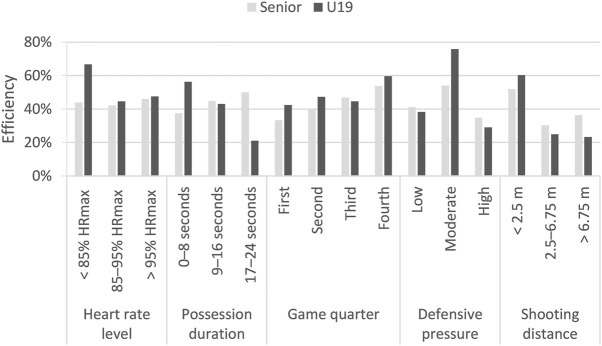
Shooting efficiency according to performance and game indicators.

Table 3 and Table 4 show the frequency distribution of each independent variable for the shooting efficiency. The tables also include the relationship between the variables. In the U19 category, the relationship between shooting efficiency and heart rate level and shooting efficiency and game quarter is not statistically significant. A statistically significant relationship was noticed between shooting efficiency and possession duration (*p* = 0.01), defensive pressure (*p* < 0.001), and shooting distance (*p* < 0.001). Players with time to end the offense, under high physical pressure and from a long distance (>6.75 m), achieved the lowest shooting efficiency. However, the players had the highest efficiency of the shooting under moderate physical pressure (Figure 1). In the senior category, the relationship between shooting efficiency and heart rate level, possession duration, game quarter, and defensive pressure is not statistically significant. Only the relation between shooting efficiency and shooting distance was statistically significant (*p* = 0.01).

**TABLE 3:** Frequency distribution (number of shots and their percentage share in each indicator) and shooting efficiency according to performance and game indicators in the U19 category.

Performance and game indicators		Dependend variable		Shooting efficiency	*p*-value	ES
		Made	Missed			
Heart rate level	<85% HRmax	18 (8%)	9 (4%)	66.7%	0.11	0.14
	85–95% HRmax	70 (31.3%)	87 (38.8%)	44.6%		
	>95% HRmax	19 (8.5%)	21 (9.4%)	47.5%		
Possession duration	0–8 s	63 (28.1%)	49 (21.9%)	56.3%	0.01	0.21
	9–16 s	40 (17.9%)	53 (23.7%)	43%		
	17–24 s	4 (1.8%)	15 (6.7%)	21.1%		
Game quarter	First	28 (12.5%)	38 (17%)	42.4%	0.31	0.13
	Second	26 (11.6%)	29 (12.9%)	47.3%		
	Third	25 (11.2%)	31 (13.8%)	44.6%		
	Fourth	28 (12.5%)	19 (8.5%)	59.6%		
Defensive pressure	Low	46 (20.5%)	74 (33%)	38.3%	<0.001	0.37
	Moderate	50 (22.3%)	16 (7.1%)	75.8%		
	High	11 (4.9%)	27 (12.1%)	29%		
Shooting distance	<2.5 m	88 (39.3%)	58 (25.9%)	60.3%	<0.001	0.34
	2.5–6.75 m	12 (5.4%)	36 (16.1%)	25%		
	>6.75 m	7 (3.1%)	23 (10.3%)	23.3%		

Note: *p*–level of statistical significance; ES, effect size.

**TABLE 4:** Frequency distribution (number of shots and their percentage share in each indicator) and shooting efficiency according to performance and game indicators in the senior category.

Performance and game indicators		Dependend variable		Shooting efficiency	*p*-value	ES
		Made	Missed			
Heart rate level	<85% HRmax	11 (4.4%)	14 (5.6%)	44%	0.9	0.03
	85–95% HRmax	80 (31.7%)	110 (43.7%)	42.1%		
	>95% HRmax	17 (6.7%)	20 (7.9%)	46%		
Possession duration	0–8 s	33 (13.1%)	55 (21.8%)	37.5%	0.4	0.09
	9–16 s	61 (24.2%)	75 (29.8%)	44.9%		
	17–24 s	14 (5.6%)	14 (5.6%)	50%		
Game quarter	First	23 (9.1%)	46 (18.3%)	33.3%	0.12	0.15
	Second	26 (10.3%)	39 (15.5%)	40%		
	Third	30 (11.9%)	34 (13.5%)	46.9%		
	Fourth	29 (11.5%)	25 (9.9%)	53.7%		
Defensive pressure	Low	52 (20.6%)	74 (29.4%)	41.3%	0.09	0.14
	Moderate	34 (13.5%)	29 (11.5%)	54%		
	High	22 (8.7%)	41 (16.3%)	34.9%		
Shooting distance	<2.5 m	68 (27%)	63 (25%)	51.9%	0.01	0.2
	2.5–6.75 m	20 (7.9%)	46 (18.3%)	30.3%		
	>6.75 m	20 (7.9%)	35 (13.9%)	36.4%		

Note: *p*–level of statistical significance; ES, effect size.

### Variables determining shooting efficiency

Backward stepwise selection of binary logistic regression removed insignificant predictors from the model in four steps based on the likelihood ratio test (χ^2^ = 50.42, df = 4, *p* < 0.001 and χ^2^ = 19.73, df = 4, *p* < 0.001 for U19 and senior category, respectively). The adjusted model with two independent variables classified the correct execution of shooting at 71.9% in U19 and 65.1% in the senior category. Estimation of regression model parameters in step four with explanatory variables, their standard errors, and the odds ratios is given in Table 5. Predictors that statistically significantly affect the shooting efficiency in both categories are defensive pressure and shooting distance. However, the interpretation of the estimated parameters is limited to the odds ratio regarding the reference category chosen at the beginning of the analysis. In the U19 category, the chance of ineffective shooting is 3.5 (95% CI; 1.43–8.52) times higher for high defensive pressure compared to the minimum pressure.

**TABLE 5:** Estimation of regression model parameters for shooting efficiency with explanatory variables in the fourth step.

Category	Independent variables		B	SE.	Wald	df	*p*	OR	95% CI for OR	
									Lower	Upper
U19	Defensive pressure	Low			20.52	2	<0.001			
		Moderate	-0.85	0.39	4.7	1	0.03	0.43	0.2	0.92
		High	1.25	0.46	7.57	1	0.01	3.5	1.43	8.52
	Shooting distance	<2.5 m			17.74	2	<0.001			
		2.5–6.75 m	1.53	0.43	12.79	1	<0.001	4.63	2	10.71
		>6.75 m	1.64	0.51	10.51	1	0.001	5.15	1.91	13.86
Senior	Defensive pressure	Low			9.49	2	0.009			
		Moderate	0.15	0.38	0.15	1	0.7	1.16	0.55	2.45
		High	1.16	0.42	7.64	1	0.01	3.19	1.4	7.26
	Shooting distance	<2.5 m			13.61	2	0.001			
		2.5–6.75 m	1.36	0.39	12.43	1	<0.001	3.9	1.83	8.31
		>6.75 m	1.19	0.42	7.81	1	0.01	3.27	1.43	7.52

Note: B–standardized beta weights; SE, standard error of the estimate; Wald–values of Wald’s test; df–degrees of freedom; *p*–the statistical significance of regression coefficients; OR, odds ratio; CI, confidence interval.

On the other hand, moderate defensive pressure is negatively related to shooting efficiency, which suggests that the chance for ineffective shooting is 0.43 (95% CI; 0.2–0.92) times lower for moderate defensive pressure compared to the minimum pressure. With increasing shooting distance, the shooting efficiency significantly decreases. The chance that shooting from a medium distance will be ineffective is 4.63 (95% CI; 2–10.71) times higher than a short distance. The chance of ineffective long-range shooting is 5.15 (95% CI; 1.91–13.86) times higher than short-range shooting. In the senior category, when the defensive pressure of the opponent was medium, the chances for ineffective shooting is 3.19 (95% CI; 1.4–7.26) times more likely, as it is under the low defensive pressure. Shooting efficiency significantly decreases when the horizontal distance increase. The chance for ineffective shooting is 3.9 (95% CI; 1.83–8.31) and 3.27 (95% CI; 1.43–7.52) times higher for medium and long-distance (respectively) when compared to the reference category of short distance. The Hosmer-Lemeshow goodness of fit test indicates that the difference between the observed and the expected frequencies is small and thus statistically insignificant (*p* = 0.997 and *p* = 0.89 for U19 and senior category, respectively).

## Discussion

### Heart rate level

This study aimed to determine the disruptive factors that can predict the shooting performance of female basketball players in real game conditions. The results yielded no significant association between shooting efficiency and heart rate level. However, it is important to mention that 88% in the U19 category and up to 90% in the senior category of all shooting attempts were performed at an heart rate level higher than 85% of HR_max_ as well as that the efficiency of the shooting performance progressively decreased with the increase of the heart rate level in both categories. Our finding can be compared to the results of previously published research reports (Vencúrik, 2016; Ardigò et al., 2018; Padulo et al., 2018) that found the negative influence of heart rate level on shooting efficiency. More precisely, the study by Vencúrik (2016) found that female basketball players had 60%, 37.5%, and 45.2% successfulness in shooting with HR at the level <85% HR_max_, 85–95% HR_max_, and >95% HR_max_, respectively. In a similar vein, Padulo et al. (2018) suggested that the efficiency of 2 PT shooting of male players at an heart rate level above 80% of HR_max_ (30%) was significantly lower compared to the efficiency of shooting at the heart rate level of 50% of HR_max_ (38.2%) and rest (42.3%). Subsequently, the efficiency of three-point (3 PT) shooting of male players in relation to heart rate level was investigated by Ardigò et al. (2018), and their study showed that the efficiency of 3 PT shooting was: 30% with HR above 80% of HR_max_, 36.8% with HR at 50% of HR_max_, and 46.8% at rest. Primarily, when both categories are considered while performing high-intensity movements, fatigue accumulation may negatively affect the shooting efficiency (Erčulj and Supej, 2009; Padulo et al., 2018). Fatigue could leads to a series of changes and compensatory mechanisms in biomechanical parameters (Rodacki et al., 2002), which subsequently affect shooting performance. However, a possible reason for this discrepancy might be that the HR during the female competitive games varies from 86 ± 2% to 93 ± 4% of HR_max_ (Matthew and Delextrat, 2009) and rarely exceeds 95%, which is somehow logical to perform more shots in this zone. Additionally, it means that there are more missed shots, as already shown by our study’s results. Following the above studies, we recommend that these findings should be considered by coaches when designing shooting programs.

### Ball possession

Contrary to our expectations, even though shooting efficiency decreased with the shot clock winding down in the U19 category but increased in the senior category, the results identified the possession duration as an insignificant predictor. To date, scant attention has been paid to the relationship between possession duration and shooting efficiency. Nevertheless, Gómez M. et al. (2015) classified the possession duration of more than 10 s as a significant predictor of shooting efficiency. Previously highlighted facts suggest that whenever the situation in the game allows, the basketball players should use an organized offense with controlled ball possession to increase shooting efficiency (Gómez M. et al., 2015). On the other hand, a relatively high percentage of shot efficiency and the number of attempts was recorded in the first 0–8 s of the offensive phase. This probably points to the importance of the transition phase (primary and secondary fast break), where more simple shots are performed compared to shots during the organized offense (Conte et al., 2017). Finally, the least taken shots with a difference in the number of successful attempts in the two categories were recorded during the last seconds of the offense. Namely, a ball release in the last seconds is often not a planned and organized offense, and the shot is strongly influenced by the opponent’s aggressive defense, which may lead to missed shot.

### Game quarter

The influence of game quarters on basketball performance has received much research attention. However, previous studies have disregarded when it comes to variations in performance that exist between quarters. While some authors found that physical performance was diminished with quarter progression, other authors did not report similar results (Scanlan et al., 2015; Reina et al., 2019). A possible reason for this discrepancy might be that a different methodological approach was used in these studies (Scanlan et al., 2015; Reina et al., 2019). Moreover, while they were primarily focused on variations in activity demands, none of these studies specifically had insight into variations in shooting performance.

On the other hand, the findings of the present study are consistent with Vaquera et al. (2016), who did not find a significant impact of the game quarter on the ball screen effectiveness. These findings would suggest that improving the shot efficiency can be related to the fact that players in the first quarter adapt to new situations while later due to fatigue or tactical goals they choose longer and controlled offensive plays which lead to a shot from a well-built and planned position (Scanlan et al., 2015; Vázquez-Guerrero et al., 2019). Moreover, increased shooting performance may have been influenced by the fact that in some games, there was such a significant score difference in favor of the teams being monitored before the start of the last quarter that the opponents may have lost their motivation in the defensive phase of the game, reduced physical pressure on players and thus simple shots were taken. Another possibility may be better fitness and wider rotation of players, which could have resulted in the already mentioned situations.

### Defensive pressure

Furthermore, our results showed that defensive pressure statistically significantly affected shot efficiency. The chance for an unsuccessful shot under high defensive pressure in U19 was 3.5 times higher than under low defensive pressure. In the senior category, the chance for an unsuccessful shot was 3.9 times higher when medium defensive pressure and 3.27 times higher when high defensive pressure was applied compared to low defensive pressure. Our findings are consistent with previous studies (Álvarez et al., 2009; Csataljay et al., 2013; Gorman and Maloney, 2016; Matulaitis and Grėbliūnas, 2021) showing that increasing the defensive pressure significantly reduces the percentage of shooting performance.

Moreover, offensive players are forced to modify their shot technique and adapt to the aggressive defense. Particularly, the offensive players increase the speed of the shot and the time spent in the air during the jump while changing the trajectory of the ball, which may influence the variations in shooting performance (Rojas et al., 2000; Gorman and Maloney, 2016). Interestingly, our finding demonstrated better shooting efficiency percentages during high defensive pressure were found in the U19 category, especially when Rojas et al. (2000) suggested that more experienced and skilled players are more adaptable to defense under high pressure. It is clear that the highlighted adaptations are the result of avoiding a possible block of a defensive player (Rojas et al., 2000). However, decreased accuracy simply means that players are not able to turn adaptations into a successful shot (Gorman and Maloney, 2016). It is still unclear whether this is due to insufficient development of technique, but we recommend that coaches and basketball players should implement more frequently actions with defensive pressure similar to the conditions of real games during the training sessions.

### Shooting distance from the basket

The data provide convincing evidence that increased horizontal distance from the basket is a key component of decreased shooting performance besides the defensive pressure. This finding is in line with previous research which examined the influence of different distances on shot efficiency (Miller and Bartlett, 1996; Erčulj and Supej, 2009; Okazaki and Rodacki, 2012; Gómez M. et al., 2015). More precisely, while Gómez M. et al. (2015) focused exclusively on the analysis of shot efficiency and came to the conclusion that with a change in distance, the performance also decreases, other authors analyzed changes in biomechanical parameters and adaptations that occur during shots from greater distances (Miller and Bartlett, 1996; Erčulj and Supej, 2009; Okazaki and Rodacki, 2012; Nakano et al., 2020). Increased ball release speed and decreased ball release height and angle, as well as the shooting angles of individual arm segments, may strongly affect the shooting performance from greater distances (Miller and Bartlett, 1996; Okazaki and Rodacki, 2012; Okazaki et al., 2015; Vencúrik et al., 2021a; Cabarkapa et al., 2022a; 2022c). Additionally, Nakano et al. (2020) concluded that a greater amount of energy transformed from the lower extremities to the upper limbs and joints was present during shots from greater distances; furthermore, a greater amount of energy causes a higher ball release speed and consequently leads to a more inaccurate shot. A further complication is that it seems more difficult for female and young basketball players (who were the target population of our research) to adapt to new situations and movement modifications with an increase in shooting distance from the basket (Erčulj and Štrumbelj, 2015).

## Limitations

Some limitations must be acknowledged, even though this is the first study that provides knowledge about factors that significantly affect shooting efficiency during official games in female basketball players belonging to different age categories and playing levels. Our dataset was limited to the number of participants and gender. These findings may not apply to professional or young male basketball players. Although the participants were members of the highest ranked teams in the Czech Republic and some of them were members of national selection teams, another limiting factor may be the small number of participants included in our study. Authors are aware that monitoring eighteen players (ten in U19 and eight in senior level) during eleven official games cannot be generalized to a wider range of female basketball players.

Finally, HR measurement is a valid and reliable way of measuring internal load (Buchheit, 2014; Schneider et al., 2018). Furthermore, more complete results would be obtained if we used other parameters such as: maximum oxygen consumption (VO_2_max), blood lactate or the Rate of Perceived Exertion (RPE). More precisely, HR can be influenced by factors other than just the intensity of the game, but due to the real game situations, it was impractical to use the additional equipment. However, the novelty of this study lies in identifying factors that affect shooting performance in the real-game conditions of female basketball players, but there is a need for further research in this area of investigation. Future studies will have to explore factors that affect shooting performance on a larger sample of participants of different ages, gender, and level of playing. Also, other contextual factors should be included in the analysis, such as: playing positions, current game score, the final result of the game, standings of the team, and opponent’s defensive system. Finally, more detailed information will lead to more specific recommendations for the training process and better outcomes during the official games.

## Conclusion and practical application

The findings of the present study indicate that defensive pressure and shooting distance significantly affect shooting efficiency. At high defensive pressure, the shooting successfulness was lower than the low defensive pressure. Shooting efficiency was lower from medium and long distances compared to short distances. The results of this study demonstrated the importance of some factors that should be given greater attention in the training process, which could increase the shooting performance of the players: 1) the frequency of shooting from different distances from the basket should be balanced; 2) shooting should be improved even under moderate and high defensive pressure; 3) the heart rate level should be taken into account when designing shooting programs, even though no significant impact on its successfulness has been demonstrated (88% in the U19 category and up to 90% in the senior category of all shooting attempts were performed at an intensity above 85% of HR_max_).

## Data Availability

The original contributions presented in the study are included in the article/Supplementary Material, further inquiries can be directed to the corresponding author.
